# Solar Salterns and Pollution: Valorization of Some Endemic Species as Sentinels in Ecotoxicology

**DOI:** 10.3390/toxics11060524

**Published:** 2023-06-10

**Authors:** Wassim Guermazi, Neila Annabi-Trabelsi, Genuario Belmonte, Kais Guermazi, Habib Ayadi, Vincent Leignel

**Affiliations:** 1 Laboratoire Biodiversité Marine et Environnement (LR18ES30), Université de Sfax, Sfax CP 3000, Tunisia; wassim.guermazi@fss.usf.tn (W.G.); neila.trabelsi@isbs.usf.tn (N.A.-T.); guermazikais90@gmail.com (K.G.); habib.ayadi@fss.usf.tn (H.A.); 2 Laboratory of Zoogeography and Fauna, University of the Salento, 73100 Lecce, Italy; genuario.belmonte@unisalento.it; 3 Laboratoire BIOSSE, Le Mans Université, Avenue Olivier Messiaen, 72000 Le Mans, France

**Keywords:** solar salterns, biota, pollution, bioremediation, sentinel species

## Abstract

Solar salterns and salt marshes are unique ecosystems with special physicochemical features and characteristic biota. Currently, there are very few studies focused on the impacts of pollution on these economic and ecological systems. Unfortunately, diversified pollution (metals, Polycyclic Aromatic Hydrocarbons, etc.) has been detected in these complex ecosystems. These hypersaline environments are under increasing threat due to anthropogenic pressures. Despite this, they represent a valuable source of microbial diversity, with taxa displaying special features in terms of environmental remediation capacities as well as economical species such as *Artemia* spp. (Branchiopoda) and *Dunaliella salina* (Chlorophyta). In this review, we discuss the impacts of pollution on these semi-artificial systems. Therefore, we have indicated the sentinel species identified in plankton communities, which can be used in ecotoxicological investigations in solar salterns. In future, researchers should increase their interest in pollution assessment in solar salterns and salt marshes.

## 1. Introduction

**Figure 1:**
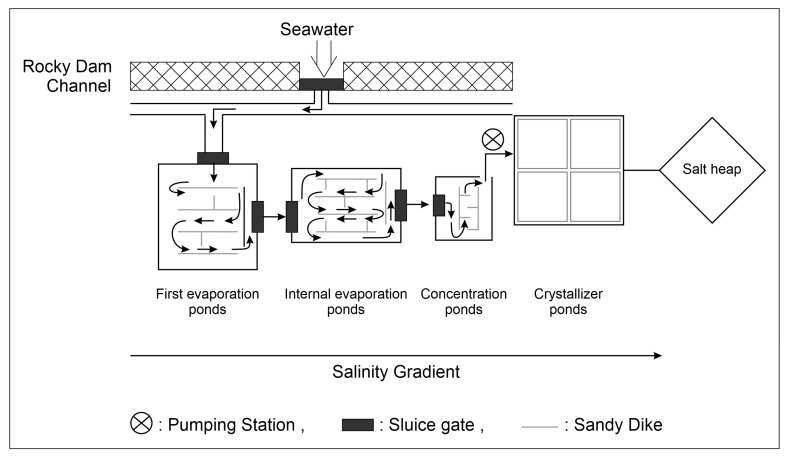
Water flow in solar salterns: the direction of the water circulation is indicated by the arrows.

Solar salterns have considerable economic, ecological, and scientific value. They are distributed globally along tropical and subtropical coasts in arid and semiarid regions [1,2]. They are mainly distributed in Mediterranean regions, where the climate is characterized by long dry periods in the summer, during which evaporation of seawater in ponds is accentuated [3]. Multi-pond salterns are human-controlled semi-artificial coastal systems designed to harvest NaCl from seawater for human consumption. In this system, seawater is pumped through a series of separate shallow ponds (Figure 1) that are typically less than 0.5 m in depth [4], in which it is gradually driven to ponds of greater salinities, ranging from seawater to sodium chloride saturation and sometimes even beyond [5]. Salterns are well known as continuous or semi-continuous systems because each set of ponds is characterized by a distinct range of salinity and biogeochemical attributes [6,7]. These thalassohaline environments are operated in repeated cycles of feeding with natural saltwater, increasing salt concentration due to water evaporation, and, finally, salt precipitation. Hence, they have certain attributes of semi-closed chemostats [6].

## 2. Physicochemical Parameters

Salterns are hypersaline extreme ecosystems with unique abiotic features, including a wide range of salinities, low oxygen, and intense ultraviolet radiation [1,7]. The physicochemical properties of seawater change due to evaporation in the flow-through multi-pond system. Although the pH of seawater is slightly alkaline, owing to carbonate buffering systems, the pH of saline water in salterns is generally close to neutral in ponds with biota [3,8,9,10,11,12]. The pH of ponds in salterns can be regulated via the salinity, temperature, and amount of carbonate ions [2]. It was shown to gradually decrease, along the distinct ponds, from 8.3 for seawater to 5.8 for magnesium chloride solutions as measured in the Sfax solar saltern in Tunisia [13] and exhibits little seasonal variation [14]. The nutrient concentrations found in solar salterns depend on a variety of parameters. Geographic factors influencing nutrients include the proximity to rivers, urban pollution, the nutrient status of the incoming seawater, and climate change [15]. The nature and extent of the fauna and flora, the season of productivity, and management practices also influence nutrient concentration [15]. According to Kobbi-Rebai [16], the internal recycling processes such as the release from sediment and the mineralization of organic matter are the key drivers of phosphorous concentrations in ponds. However, these concentrations are mainly impacted by the seawater inflow in the first pond [16]. Total phosphorus (TP) would be one of the most suitable chemical parameters from which to propose a methodology for the determination of trophic status in solar salterns [17]. Among solar salterns, both oligotrophic [18,19] and eutrophic systems [11,20,21,22,23,24] have been described. However, nutrients are concentrated in first ponds and decrease with increases in the salt concentration [22]. Nitrate and nitrite concentrations are influenced by halophilic bacteria activity [25]. Extremely halophilic denitrifying bacteria in hypersaline environments reduce nitrate to nitrite, nitrous oxide, and even dinitrogen [26].

## 3. Biota

**Figure 2:**
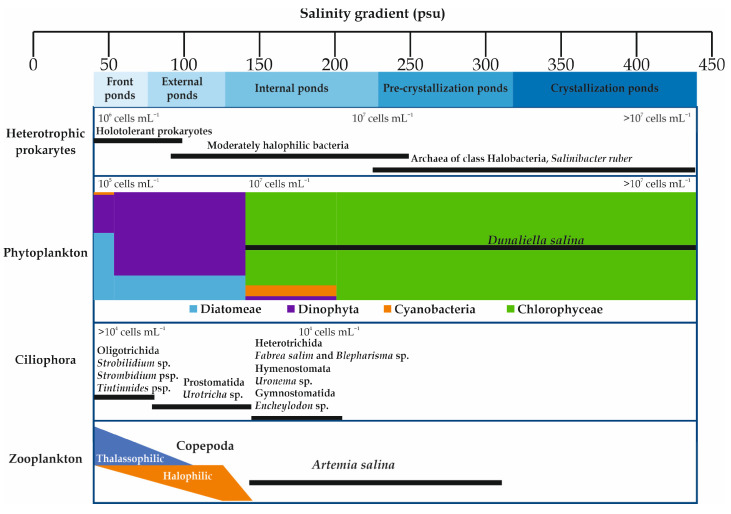
Distribution of the dominant living beings (plankton and heterotrophic prokaryotes) along the salinity gradient in solar salterns. Data compiled from and drawing based on [9,11,16,43,44,48,49,53,58].

The biota of the salterns are not only of great scientific interest, but also have a direct impact on the quality and the quantity of the salt produced via evaporation and precipitation [6,27]. Solar salterns are inhabited by highly specialized extremophiles (Figure 2). Many ecological changes happen throughout the salinity gradient. Biodiversity decreases with the increase in salinity [9,11,28,29,30,31], with the zonal biological community structure ranging from marine to extreme halophilic communities [9,32,33,34]. Archaea and Bacteria are the two components of the microbial community in solar salterns [28,35,36]. These organisms are generally members of the archaeal Halobacteria class and in the bacterial family of Salinibacteraceae (Rhodothermia class) [37,38]. Nevertheless, relatively high species richness is noted within each class [39]. Analysis of prokaryotic communities’ compositions in solar salterns in Mexico, Spain, Tunisia, and Turkey showed that the most highly represented genera were *Haloquadratum*, followed by *Halorubrum*, *Haloarcula*, and *Halonotius* [40,41,42,43]. In the crystallizer ponds, which have very low prokaryotic diversity with Archaea making up the dominant fraction [44,45], the most abundant Archaea observed were *Haloquadratum walsbyi* and *Halorubrum* sp. [5]. *Salinibacter* phylotypes and other Bacteroidetes are highly abundant in the microbial community [44,46,47]. However, in lower salinity ponds, a more diverse assemblage of Archaea and Bacteria was detected [47].

The phytoplankton community of *Dinophyta* and Diatomeae found in lower salinities was replaced by a community of *Dunaliella* and Cyanobacteria adapted to higher salt concentrations [9,11,22,48,49,50]. A shift was observed from Diatomeae to Dinophyta dominance along the salinity gradient (from 38 to 86 psu), as exemplified in the Sfax solar saltern in Tunisia [51]. This salinity gradient was negatively correlated with the amount of Diatomeae, while it was positively correlated with the amount of Dinophyta [51]. The Chlorophyta *Dunaliella salina* and the Cyanobacteria *Aphanothece* sp. and *Phormidium* sp. dominated the phytoplanktonic community in the saltiest ponds from 190 to 476 psu [9]. *Dunaliella salina* is the most ubiquitous eukaryotic microorganism in hypersaline environments [15]. The phytoplankton community in the crystallizer ponds (TS > 300 psu) was entirely composed by *D. salina* (for the Sfax solar saltern, see [9,30]). *Dunaliella salina* release enzymes and nitrogen compounds into the water which favor the growth of halophilic bacteria and, in turn, accelerate evaporation [31]. According to Elloumi et al. [9], the Ciliophora community is dominated by Oligotrichida (*Strobilidium* sp., *Strombidium* sp., *Tintinnides* sp.) at salinities from 41 psu to 46.9 psu. The Prostomatida *Urotricha* sp. became the dominant taxon at salinity values of 45.6–146.8 psu, but from 184 to 203.2 psu the Ciliophora shifted to a dominance of Heterotrichida (*Fabrea salina* and *Blepharisma* sp.), Hymenostomata (*Uronema* sp.), and Gymnostomatida (*Encheylodon* sp.).

Among zooplankton groups, Artemiidae (Branchiopoda) and Copepoda are the most abundant groups in hypersaline ecosystems [14,52,53,54,55,56]. In keeping with their fluctuations in abundance along the salinity gradient in the Sfax solar saltern, Copepoda species are split into thalassophilic species (species whose abundance decreases with increasing salinity) and halophilic species (species whose abundance increases with increasing salinity) [12,16,34,51,56]. At salinities from 157 to 312 psu, the euryhaline *Artemia* spp. is the only zooplanktonic taxon [14,54,56,57].

## 4. Pollution Diversity in Solar Salterns

Hypersaline environments, including solar salterns and salt marshes, are often polluted with various contaminants [59,60]. They are fragile econiches and are very susceptible to disturbances [61]. Knowledge of pollution existing in solar salterns is not generalizable to every site and the completion of analyses of a great number of sites remains to be achieved (Table 1).

The metal (Cd, Cu, Pb, and Zn) concentrations in the salt marsh sediments of the Karnaphuli River coast (Bangladesh) are related to contamination from domestic and industrial discharges [62] (Table 1). The metal concentrations in the sediments were: 105.0 ppm for Zn, 26.70 ppm for Pb, 45.79 ppm for Cu, and 0.43 ppm for Cd [62]. The Ribandar solar salterns (India) are fed by the Mandovi Estuary and are, in turn, vulnerable to metal effluent influxes from ferromanganese ore mining activity, barge traffic, and sewage disposal, affecting the water and sediment quality in the salt pan and its inhabitant organisms [63]. Sediment quality indicates that the Ribandar solar saltern sediments were moderately contaminated by Co, Fe, Mn, Ni, Pb, and Zn during the salt-making seasons [63]. The concentrations of heavy metals in the sediments ranged from a minimum of 1.7 ± 0.1–2.6 ± 0.7 ppm Pb to a maximum of 44 ± 21.6–62.8 ± 23.6 ppm Zn [63] (Table 1). 

In the Pomorie brine salterns (the Black Sea, Bulgaria), the distribution of Pb and Cu in the three constituents of the brine system, the salt solution, colloidal particles, and biota (*Halobacterium salinarium* and microalgae *Dunaliela salina*), showed the highest percentages in biota, at 45% and 48%, respectively [64]. However, Cd and Bi have not been detected in biota and are uniformly distributed between the salt solution and colloidal particles [64] (Table 1).

A comparison of the concentrations of trace metals in the sediments of three salt marshes (Tinto, Odiel, and Piedras) in Huelva, Spain showed that the Tinto sediments were the most polluted, with high amounts of As (600 ppm), Cu (3300 ppm), and Zn (2500 ppm) [65] (Table 1). On the other hand, the Piedras estuary had not been affected by anthropogenic inputs and was the least polluted estuary [65].

As an example of pollution and dysfunction in the hypersaline ecosystem, we present the case of the Sfax solar saltern in Tunisia. Since the 1950s, there has been rapid industrialization and urbanization along the coastal area near the solar saltern. Thus, the treated domestic wastewater (pH 8.3) and the untreated industrial wastewater released diversified pollutants. For example, near the Sfax solar saltern (Tunisia), the phosphate treatment plant, SIAPE, and the gypsum water produced from the lixiviation of SIAPE phosphogypsum deposits are sources of heavy metals which are rapidly precipitated in marine sediments [66,67,68,69]. Such a mixture of effluents, which has a pH of 3, facilitated the dissolution of heavy metals, thus enhancing the movement of the bioavailable ionic forms of these metals in seawater.

The brine metal concentrations in the Sfax solar saltern vary from 0.065 to 1.57 mg L^−1^ for Zn, 0.002–0.034 mg L^−1^ for Cd, 0.006–0.064 mg L^−1^ for Cu, and 0.002–0.128 mg L^−1^ for Pb. The analysed metals are present in the following order: Zn > Pb > Cu > Cd. The computed enrichment factors (EFs) showed significant brine contamination due the impact of industrial particulate fallout highly enriched with heavy metals [70]. In fact, concentrations of trace metals in surface sediment samples have shown Fe varying from 8750 to 8889 ppm of dry weight, Zn from 39.92 to 574.89 ppm of dry weight, Pb from 18.98 to 233.46 ppm of dry weight, Ni from 17.47 to 160.92 ppm of dry weight, Cu from 13 to 98 ppm of dry weight, and Cd from 4.86 to 37.42 ppm of dry weight [69]. The highest metal concentrations have been found in sites frequently subject to local pollutant sources and in sites often saturated by high-tide marine water which drains industrial waste from the port area. This agrees with the findings of Amdouni [71], who reported the presence of a variety of metals (Al, Cd, Cu, Pb, and Zn) in crystallization ponds.

According to Amdouni [72], the concentrations of trace elements in brines are affected by the evaporation phenomenon in the same way as those of the major elements. Initially, the evaporation effect is very limited and the behavior of trace elements is under the direct influence of the biological activities which colonize the first ponds of the saline saltern. Thus, the evolution of trace element concentration seems to be controlled only by the evaporation–salt precipitation antagonist effect in the more concentrated brine where biological activity is absent or very limited. However, the concentrations of trace metals such as mercury, copper, zinc, lead, and cadmium in cysts and biomasses of *Artemia* (Branchiopoda) originating from the Sfax solar saltern are lower than those recorded in other strains, i.e., those from other localities that are already commercialized and used in larval fish feeding [73]. The lowest bioaccumulation of trace elements in *Artemia* was observed at the highest salinity (190 psu) [74]. In fact, the bioavailability of elements often decreases with increasing salinity due to trace element complexation [75]. Compared to studies carried out at various solar salterns around the world, the presence of Ni seems to be a characteristic novelty, and the order of abundance of the metal concentration is not entirely similar either [62,63]. 

Solar salterns and salt marshes may be also contaminated by aliphatic and aromatic hydrocarbons. A hydrocarbon analysis performed in the Sfax solar saltern allowed for the detection of aliphatic hydrocarbons and n-alkanes [76]. The total aliphatic hydrocarbon concentrations varied from 92.5 mg. L^−1^ in the first pond, which has marine characteristics, to 661.1 mg. L^−1^ in the crystallizer pond [76]. The use of n-alkane distribution indices coupled with environmental factors permitted for the assertion of the assumption that a major proportion of the hydrocarbons resulted from eukaryotic and prokaryotic communities and a low proportion of the hydrocarbons might be petrogenic [76]. Hydrocarbon extraction and analysis from the Sfax coastal region near the solar saltern showed that the sediments are contaminated by petrogenic aliphatic and aromatic hydrocarbons [77,78,79]. The transportation of oil, shipping and industrial activities, urban runoff, and waste water discharge are the main sources of hydrocarbon contamination in the Sfax coastal zone [77].

**Table 1:** Contaminants recorded in solar salterns.

Contaminants	Solar Saltern	Fraction	References
Trace metals	Sfax solar saltern (Tunisia)	Surface sediments	[69,80]
		Water	[70,72]
		Biota: *Artemia salina*	[73]
	Ribandar solar saltern (India)	Surface sediments	[63]
	Porteresia Bed, Karnafully coastal area (Bangladesh)	Surface sediments	[62]
	The Black Sea brine Pomorie salterns, Burgas (Bulgaria)	Water	[64]
		Biota *Halobacterium salinarium* and microalgae *Dunaliela salina*	[64]
		Colloidal particles	[64]
	Tinto, Odiel, and Piedras salt marshes in Huelva (Spain).	Surface sediments	[65]
Hydrocarbons	Sfax solar saltern (Tunisia)	Surface sediments	[76]
		Water	[76]

## 5. Solar Salterns: Potentialities for Bioremediation

Solar salterns act as ecological sinks with the potential to transform native bacterial populations into metal-resistant strains corresponding to the dynamic changes in the surrounding metal concentrations [74]. *Dunaliella salina* (microalgae) isolated from the Sfax solar saltern (Tunisia) also shows a high capacity for metal absorption [75]. Metallothioneins and its secondary metabolites provide a protective role against the toxic effects of metals and protect cells against oxidative stress [75,76]. *Halobacterium salinarum* isolated from the Sfax solar saltern also displays a significant tolerance for heavy metals and has the potential for the successful biotechnological use for the bioremediation of heavy metal-contaminated environments [77]. Syed and Chinthala [78] reported that the *Bacillus* sp. from Indian solar salterns were able to remove up to 90% of Pb from aqueous solutions.

Hydrocarbons are also present in hypersaline ecosystems, where it can also be biodegraded by solar saltern-indigenous hydrocarbon-degrading halophilic organisms [60,81,82]. Erdoğmuş et al. [83] showed that naphthalene, phenanthrene, and pyrene can be degraded by several archaeal strains, including *Halobacterium piscisalsi*, *Halorubrum ezzemoulense*, *Halobacterium salinarium*, *Haloarcula hispanica*, *Haloferax* sp. and *Halorubrum* sp. isolated from brine samples from the Camalt saltern in Turkey. These same genera are present in the Sfax solar saltern [40,45,58,84] and other salterns around the world [85,86,87,88].

## 6. Sentinel Species

Solar salterns are facing multiple anthropogenic pressures, such as eutrophication and pollution, combined with climatic fluctuations, such as global warming [23,61], that challenge the development of ecological indicators. Some species may also serve as useful sentinels for monitoring pollution in the solar saltern environment. An optimal sentinel should have a measurable response (including the accumulation of tissue residues) and sensitivity to the agent or class of agents being considered [89,90]. Moreover, the selected sentinel should be ubiquitous in solar salterns.

Plankton is a suitable group in which to find various ecological indicators at different levels of organization (individual, population, and community). The relationship between the individual and population levels can be discovered by studying the variability in the lifetime traits of sentinel species [91]. Ideally, plankton, having a basal position in the trophic chain, represent key information about the structure, function, and/or composition of solar salterns. In this review, we target *Dunaliella salina* (Chlorophyta), *Artemia salina*, and *Tisbe battagliai* (Crustacea), which are widely distributed species in solar salterns and have fast growth rates and short generation lengths. All of these characteristics allow them to function as good indicators of natural and/or anthropogenic variabilities in solar salterns and salt marshes. The life cycle properties of these species were studied in the laboratory under controlled natural conditions [92,93,94]. *Dunaliella salina*, *T. battagliai*, and *A. salina* are used regularly for the toxicity testing of various chemicals [95,96,97,98]. 

*Dunaliella* spp. (Chlorophyta) possess the unique and remarkable ability to survive in extreme conditions, such as a wide range of NaCl concentrations, intense light, high temperatures, and broad pH values from 1 to 11 [99,100,101,102]. This species of microalgae is a major constituent of all-natural hypersaline environments and one of the most salt-tolerant life forms [34,103,104,105]. It is also adapted to very high levels of ultraviolet (UV) radiation. It is thought that its carotene content functions as a sunscreen, protecting chlorophyll and DNA from harmful UV irradiation [105]. Thus, this microalga is widely used as a model system for studying the response to stress [106] and as a biological indicator of different environmental pollutants [107]. For example, trace metals can induce oxidative stress in *D. salina* by generating reactive oxygen species (ROS) such as hydroxyl radical (•OH), superoxide anion (O2•−), singlet oxygen (O2*), and hydrogen peroxide (H_2_O_2_) via auto-oxidation, blocking essential functional groups in biomolecules, or substituting essential metal ions [108,109,110]. Generated ROS could interact with lipids, proteins, and nucleic acids, resulting in their degradation [111] and in membrane instability and photobleaching of their photosynthetic pigments, thus limiting photosynthesis efficiency and growth [112]. In fact, when exposed to Cd, the growth rate of *D. salina* dropped by 51.44–59.33%, indicating an inhibition in cell growth with a reduction in lipid content [113]. According to Belghith et al. [114], during the latency phase (day 3), the growth of *D. salina* treated with Cd showed an inhibition that varied between 26.26% for a concentration of 25 mg L^−1^ and 58.64% for a concentration of 150 mg L^−1^. The total chlorophyll in this microalga decreased with an increase in CuCl_2_ [115]. A concentration of Nickel of 0.5 mg L^−1^ caused a shift in the fatty acid profile towards saturated fatty acids (C14:0, C16:0, C20:0) in *D. salina* with an upshift of C22:0 behenic acid [116]. To cope with this critical situation, *D. salina* cells may produce antioxidant compounds such as the pigments or enzymes of superoxide dismutase and catalase, which are responsible for quenching ROS [117]. Widowati et al. [118] showed that the high antioxidant activity of D. salina. β-carotenoids acts as a supportive antioxidant. Humic acid was reported to protect D. salina cells against Ni^2+^ stress by means of forming humic acid–Ni^2+^ complexes and/or by adsorbing on the cell surface, and thus creating an additional barrier for Ni^2+^ uptake [116]. Cd and Pb treatments induced an increase in the expression level of metal chelator synthesis as a further defense mechanism against oxidative stress in *D. salina* [113]. In fact, its relatively unique ability to accumulate glycerol and β-carotene in response to osmotic stress has made D. salina an ideal model organism for dissecting the molecular mechanism(s) of osmotic stress responses [106]. *Dunaliella salina* has high tendency to accumulate Cd, Pb, and Zn [119,120] and has shown an increase in uptake of Zn with an increasing concentration of metal ions in its medium [119]. This organism lacks also a rigid cell wall and can be used as a model marine organism [121]. Thus, this microalga has an obvious applicable value as a test organism in the research field of environmental toxicology [98,107,122,123].

Due to its tolerance to salt concentration, *Artemia* spp. (Branchiopoda) is an important biological indicator for hyperhaline environments of the marine salt water type [124,125]. This microcrustacean is characterized by the standard features of organisms with short life cycles: easy to culture, readily available, highly adaptable to adverse environmental conditions, small body size, high fecundity, bisexual/parthenogenetic reproduction strategy, adaptability to varied nutrient resources as it is a non-selective filter-feeder, and offers a low-cost bioassay of toxicity, quick results, and non-aseptic technique compatibility [124,125,126,127]. In adverse conditions, it produces resting cysts which hatch by rehydrating in saltwater, even after several years [128,129,130]. This model species is recommended for use by the US Environmental Protection Agency as one of the acute toxicity testing species [131]. Several endpoints can be considered with *A. salina*, including short-term (24–48 h) and long-term (14 days) mortality, cyst hatchability, biomass productivity, biomarker expression/inhibition, and bioaccumulation on larvae, as well as the organisms’ reproductive abilities [132]. Behavioral responses are also sensitive biomarkers for *Artemia* [133,134]. The toxicity of trace metals has been tested using *A. salina* as a standard reference over the last few decades [135,136,137,138,139,140,141,142]. It has been selected as an organism for bioassay because of its ability to accumulate trace metals and thereafter transfer them to higher levels [143]. Thus, [135,144] propose the nauplii of *A. salina* as a good bioindicator for trace metal contamination. Kokkali et al. [137] proposed using the swimming speed of *A. salina* as a parameter which is affected by the contamination and biochemical damage induced by metals in marine environments. The trace metal accumulation in *Artemia* nauplii increased linearly with an increase in the heavy metal concentration in water. The metals could be detected in nauplii exposed to a single heavy metal at dosages of 5 μg L^−1^ for Cu, Fe, Hg, Mn, and Zn; 25 μg L^−1^ for Cd and Cr; 50 μg L^−1^ for Co; 100 µg L^−1^ for Ni; and 250 μg L^−1^ for Pb [145]. The hatch rate of *Artemia* eggs is reduced to 50% (from 1/15 to 1/30 of the LC_50_ 48h) and is more sensitive to metals when compared to Artemia nauplii [135]. Thus, *Artemia salina* is an excellent sentinel for metal pollution. Antioxidant enzymes and metallothioneins are good biomarkers for measuring biological responses to specific metal exposure, owing to their ease of measurement. For example, Cd exposure increased the activity of the antioxidants CAT, GPx, and SOD and the concentration of metallothioneins (23.9 ± 0.9 ng/100 mg) [146]. Nevertheless, despite its current practical use, several criticisms exist against the use of *Artemia* as model organism. Persoone and Wells [147] proposed a summary of the main reasons:

i. the presence of *Artemia* in high-salinity ecosystems indicates that it is not competitive with other zooplankton;
ii. it probably lacks sensitivity to the actions of some contaminants due to its high tolerance to salinity.

Harpacticoida have been selected for use in toxicity tests based on their ecological importance as a prey source for many marine consumer species, their widespread occurrence in marine waters, and their biological features (small size, short life cycle, and ease of culturing) [148,149,150,151]. *Tisbe battagliai* is sensitive to a variety of environmental contaminants and is particularly amenable to laboratory testing [152,153,154,155,156,157]. ISO [158] recommends T. battagliai for acute standard toxicity bioassays. He is observed in the Tunisian solar salterns in Sfax [16] and Sousse [55]. In the Sfax solar saltern, an abundance of *T. battagliai* correlated positively with nutrient content [16]. It is spread along the coast of Sfax and is well adapted to and tolerant of coastal anthropogenic inputs [159]. Populations of *T. battagliai* can be harvested any time of the year in all their different life stages from the front ponds of the Sfax solar saltern. It is easy to find individuals for use in several types of bioassays, like the copepodid stage for acute testing or nauplii that are less than 18 h old for developmental tests [151]. 

## 7. Biosensors and Biotests

Monitoring of the water and sediment qualities of solar salterns is crucial. Biological methods for quality assessment have developed substantially. Such methods are increasingly incorporated with technological solutions, i.e., the use of biosensor methods. Biosensors are analytical devices which utilize the sensitivity and selectivity of a bio-receptor attached onto the surface of a physical transducer. The transducer is able to respond to and transform a biochemical and/or physicochemical property into a measurable signal as a result of a biorecognition event between the bioreceptor and its target analyte [160]. Biotests are based on using living organisms that react in a specific way to pollution with metal, organic, and biogenic compounds. The analyses are conducted in laboratories rather than in the field [161]. *Artemia salina* larvae are used in a test which consists of determining the number of organisms in the lethal stage in a given water sample. The test is carried out in salt water for 24 h. Dead organisms are counted using a magnifier [162]. *Artemia salina* may be used as a suitable biosensor instead of carrying out expensive tests on higher vertebrates [163]. A biotest using *A. salina* to monitor the cumulative effects of Cd, Cu, Pb, and Zn in sediments has been published [164]. Dvořák et al. [163] demonstrated the possibility of using *A. salina* for biotests of the second generation as a sensor for the co-exposure of a number of agents at various concentrations, such as trace metals and ionizing irradiation. The SiO_2_@NBDBIA material is used as biosensing probe for oxoanions (CrO_4_^2−^, Cr_2_O_7_^2−^, and MnO_4_^−^) in the living organism *A. salina* through fluorescence imaging [165]. SiO_2_@NAPIA was used to detect ferric ions in tap water samples via biosensing of ferric ions in *A. salina* through fluorescence imaging [166]. Aeruginic acid (H_2_L), a weakly fluorescent natural ionophore, can be synthesized via one-pot mechanochemical grinding [167]. This natural ionophore, which is fully water soluble, non-toxic, and permeable in nature, can visualize Zn^2+^ in the gastrointestinal tract of live whole brine shrimp *Artemia* [167]. These tests offer new possibilities for the toxicity testing of various environmental agents and their combinations at low concentrations in solar salterns. Such alternative biotests cannot fully replace conventional tests in a laboratory setting, but they have the potential to markedly reduce the number and extent of such experiments.

## 8. Conclusions

Solar salterns are unique ecosystems with special physicochemical features and characteristic biota. They represent a valuable source of microbial diversity with taxa displaying special features in terms of environmental remediation capacities. These hypersaline environments are threatened by anthropogenic activities like pollution by trace metals and hydrocarbons, indicating the vulnerability of these ecosystems and their area of ecological relevance. Special attention should be directed to management of these fragile ecosystems. For example, constant water and sediment monitoring must be considered to avoid the dissemination of contaminants in the salterns and to protect the purity of the salt extracted. The individuation of sentinel species in the plankton community can enrich ecological and ecotoxicological studies in solar salterns. For example, *A. salina* (Branchiopoda), *D. salina* (Chlorophyta), and *T. battagliai* (Copepoda) can be promoted as good candidates for the role of sentinel species in solar salterns. It may be interesting to define other species which may be used as sentinels occupying distinct ecological niches in solar salterns or salt marshes to study the effects of pollutants accumulated in these ecosystems.
